# Investigation on the Structural Integrity of Solid Propellant Grains with Different-Sized Void Defects

**DOI:** 10.3390/ma19143151

**Published:** 2026-07-22

**Authors:** Jianru Wang, Kai Liu, Tuanwei Xu, Jinkang Du, Yuanzhe Liang, Wenjing Li, Peng Cao

**Affiliations:** 1 Academy of Aerospace Solid Propulsion Technology, Xi’an 710025, China; wjr104zah@126.com; 2 National Graduate College for Elite Engineers, Beihang University, Beijing 100191, China; 15509188271@163.com; 3 The Institute of Xi’an Aerospace Solid Propulsion Technology, Xi’an 710025, China; xutuanwei525@163.com (T.X.); dujinkang@nuaa.edu.cn (J.D.); 18810820456@163.com (Y.L.); 4 National Key Laboratory of Solid Rocket Propulsion, Xi’an 710025, China; 5 College of Architecture and Civil Engineering, Beijing University of Technology, Beijing 100124, China; wenjingli1022@126.com

**Keywords:** void defect, solid propellant, propellant grain integrity, engineering design parameters

## Abstract

During the service of solid rocket motors, propellant grains need to bear various loads such as curing cooling, gravity, and combustion internal pressure. The internal pore defects will seriously affect the structural integrity. In this paper, a three-dimensional finite element model of propellant–insulation layer–mold is established to study the structural responses of pore defects with different sizes (30–100 mm) under three typical working conditions: curing cooling, curing cooling coupled with gravity, and internal pressure loading. It is found that under the curing cooling condition, compared with the non-porous propellant grain structure, the structure with pores will raise the overall mechanical response of the propellant grain, and the maximum stress and strain are mainly concentrated in the front end of the core hole and the wing groove area. The pore size has a limited impact on the overall stress distribution, but will change the local stress concentration degree. Among them, the 80 mm pore reduces the stress in the wing groove area through stress field interference. Moreover, large-size pores will significantly weaken the structural bearing capacity and increase the contact pressure between the propellant and the core mold. Under the condition of curing cooling coupled with gravity, the stress and strain are mainly distributed at the edge of the pores, and the values increase with the increase of pore size. Under the action of internal pressure load, the stress and strain in the middle section of the propellant grain have no obvious change, but stress concentration occurs in the transition area between the core hole and the wing groove and at the end of the wing groove. The results of this study provide a reference for the integrity evaluation and structural optimization of propellant grains with pore defects.

## 1. Introduction

Solid rocket motors (SRMs) are widely utilized in strategic and tactical missiles, aerospace launch vehicle boosters, and various rapid-response flight vehicles due to their structural simplicity, high reliability, rapid response, and capacity for long-term storage [1,2,3]. As the primary energy source for these motors, solid propellants are cast into fixed grain configurations during manufacturing. This eliminates the need for complex propellant loading and delivery systems, thereby significantly enhancing the rapid-reaction capabilities and operational safety of the power system [4,5]. However, the mechanical properties of solid propellants vary significantly across a wide temperature range, and their viscoelastic response is strongly dependent on temperature and loading rate [6,7]. Furthermore, during their service life, propellant grains must endure complex loading conditions, including thermal stress, gravitational loads, and internal combustion pressure [8,9]. Consequently, maintaining the structural integrity of the propellant grain remains a critical focus in the design and reliability assessment of SRMs [10,11].

Solid propellants are composite materials consisting of a binder matrix embedded with oxidizer and metallic fuel particles [12,13]. This complex internal structure results in characteristic viscoelastic behavior and large-deformation properties. Preserving the structural integrity of the grain is vital for motor safety. Internal structural failures—such as debonding, micro-cracking, or macro-fractures—can lead to ignition failure or an abnormal increase in burning rate during operation, potentially causing catastrophic combustion instability or explosions [14]. Among various structural flaws, porosity is a common initial defect. These internal pore defects primarily stem from the inherent material properties of the propellant and the manufacturing process. During the casting phase, micro-bubbles may be entrained during mixing, which remain as residual pores after the propellant solidifies. Furthermore, uneven volumetric shrinkage during the chemical cross-linking process can create internal voids or cracks. Differences in chemical properties between the solid particles and the matrix also make the interfaces susceptible to stress-induced debonding, forming micro-voids that can expand into larger pores as stress increases. Similarly, complex mechanical loads or tensile stresses can further induce the formation of cracks and pores [15]. The presence of porosity significantly impacts the mechanical performance, combustion characteristics, and overall safety of the solid rocket motor. Under ignition pressurization, severe stress concentration occurs around these pores, greatly weakening the propellant’s mechanical strength. Stress at the pore tips can trigger the initiation and propagation of micro-cracks, ultimately leading to grain cracking or structural failure [16]. As the depth-to-width ratio of these pores increases, both the internal pressure and the local gas flow velocity within the combustion chamber rise. This interferes with the internal flow field and pressure distribution, adversely affecting motor performance [17].

Furthermore, porosity alters local combustion behavior, causing non-uniformity in burning rates and pressure distribution. When superimposed on residual thermal stresses from the solidification process, these effects can induce local yielding or interfacial slip, threatening the structural integrity of the grain. Recent research suggests that propellant porosity is a key indicator for evaluating storage life, as high porosity and microstructural defects can accelerate material aging and negatively impact long-term service performance and durability [18,19]. Studies utilizing Representative Volume Elements (RVE) and Cohesive Zone Models (CZM) to simulate micro-damage evolution have shown that higher porosity accelerates internal damage and significantly reduces macro-mechanical properties [20,21,22]. Chen [18] employed finite element analysis (FEA) and X-ray Computed Tomography (XCT) to develop precise models, revealing how porosity causes uneven stress distribution and affects structural integrity during non-linear thermal viscoelastic analysis. Conversely, Wubuliaisan, M [22] found that under specific conditions (60 °C, internal pressure, and 70 g acceleration), maximum stresses in porous propellants did not exceed safety limits. Thus, investigating the impact of pore size on propellant mechanical properties is essential for enhancing reliability and providing a theoretical basis for integrity analysis. The evolution of the extrusion pressure between the grain and the mandrel is a vital parameter for optimizing the propellant curing process. This pressure results from the material’s rheological properties and the mold geometry during solidification. It is influenced by the chemical reaction rates, heat conduction, and the specific composition of the propellant. Extrusion pressure is a primary factor affecting the successful demolding of the cured propellant from the mandrel. Under this pressure, the propellant may undergo compressive deformation or shear failure at the interface. Porosity reduces the local elastic modulus and affects thermal expansion matching, leading to stress redistribution and a weakening of the effective contact pressure [23,24]. Research indicates that interfacial extrusion pressure in porous regions can be 15% to 30% lower than in dense regions [25,26]. However, there is currently limited research on how these defects specifically impact demolding.

To address these research gaps, this paper establishes numerical analysis models for solid propellant grains containing pore defects. We systematically investigate internal stress distribution and structural integrity under three typical conditions: curing cooldown, combined curing cooldown and gravity, and internal pressure loading. This study aims to provide a theoretical foundation for the structural design and reliability assessment of solid rocket motor propellant grains.

## 2. Finite Element Simulation Model

### 2.1. Finite Element Model Construction for Porous Propellant Grains

In actual solid rocket motor design, the global geometry of the propellant grain is prescribed; however, defects with different sizes may arise during fabrication or in-service operation. Under a fixed external confinement, larger defects are more likely to cause pronounced local stress concentrations, which may promote the onset and evolution of damage. Therefore, this study aims to simulate this specific condition and investigate the influence of different void defect sizes on the structural integrity of a solid rocket motor propellant grain within a component segment with fixed geometric boundaries. Meanwhile, considering that large defects may form in solid propellants during manufacturing or service due to processing issues, material degradation, or mechanical damage, void models with radii ranging from 30 to 100 mm were adopted to evaluate the structural integrity of the grain. The 30 mm void was used to represent the influence of relatively small macroscopic defects on local stress concentration. The 60 mm and 80 mm voids were selected to investigate the evolution of the stress field and load-transfer path as the void size increases. The 100 mm void was regarded as a severe large-scale defect and was used to assess the load-bearing capacity of the grain under an extreme defect condition. If the void size were further increased, the defect morphology might no longer be appropriately represented as an isolated spherical cavity, but could instead evolve into a through crack, large-area debonding, or local material loss, corresponding to different failure mechanisms [27]. These dimensions do not represent the size of individual bubbles generated during propellant blending and manufacturing, but rather account for pore defects from a practical engineering perspective. We define these defects as macroscopic imperfections at idealized engineering scales, specifically designed for void-size sensitivity analysis and conservative structural integrity assessment. The selected range of pore sizes represents extreme scenarios that may arise due to process anomalies, material properties, or mechanical damage leading to macroscopic defects in solid propellants during manufacturing or use.

A single-void model was used in this study, in which only the void size was varied while the void location, material parameters, and boundary conditions were kept constant, thereby clarifying the effect of void size on the structural integrity of the propellant grain. When multiple voids are considered at the same time, void–void interaction, random spatial distribution, and spacing effects among defects collectively affect the stress-field evolution inside the propellant grain, thereby making it difficult to isolate the intrinsic influence of void size on grain structural integrity. The void was simplified as an isolated spherical cavity, while irregular void shapes, clustered pores, multiple interacting defects, and random spatial distributions were not considered. It should be emphasized that the present results are mainly applicable to comparative assessment of void-size effects and identification of structurally vulnerable regions, while direct prediction of failure load and service life requires further experimental validation.

**Figure 1:**
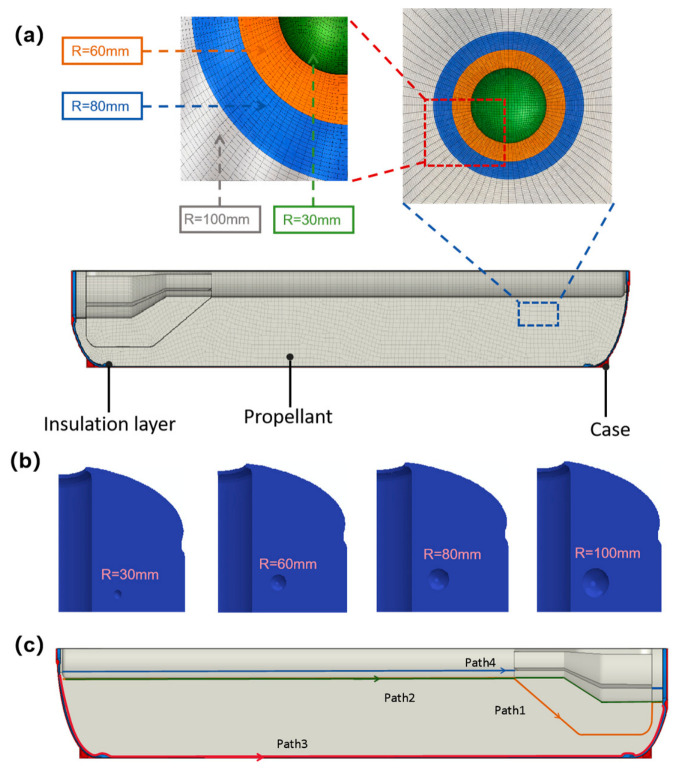
Finite element model of propellant with pores of different radii (**a**) Mesh details of voids with different radii. (**b**) Propellant grain models with void defects of different radii. (**c**) Illustration of the four defined characteristic paths.

The motor consists of four main components: the propellant grain, composite case, insulation layer, and mandrel; artificial debonding layers were arranged at both ends, and the front skirt, aft skirt, and end closures were appropriately simplified. The regions surrounding the artificial debonding layers were locally refined to improve the resolution of the numerical results. The grain was modeled as a winged-column configuration, in which eight slots were evenly arranged at the rear end and the central portion was represented by a cylindrical tubular grain section. Based on the geometric symmetry of the motor structure, a quarter-scale geometric model was established. Symmetric boundary conditions (zero circumferential displacement) were applied to the two lateral cutting surfaces of the finite element model, and a fixed boundary constraint was imposed on the front skirt. Symmetric boundary conditions (zero circumferential displacement) were applied to the two lateral cutting surfaces of the finite element model, and a fixed boundary constraint was imposed on the front skirt. Tie constraints were defined between the case and insulation layer and between the insulation layer and propellant grain, while contact was assigned between the grain bore and the mandrel to ensure deformation compatibility among different components during simulation. As shown in Figure 1c, four characteristic paths were defined: Path 1 from the front end of the inner bore to the wing-slot end, Path 2 from the front end of the inner bore to the wing-column end, Path 3 along the propellant/insulation interface on one side of the wing slot from the front to the rear end, and Path 4 from the front end of the inner bore to the wing-slot end.

### 2.2. Thermal–Chemical Model of Solid Propellant Curing

During the propellant curing process, the three-dimensional transient heat conduction governing equation is derived based on Fourier’s Law of heat conduction and the principle of energy conservation [28]:

(1) $$\rho C_{p}\frac{\partial T}{\partial t}=\frac{\partial T}{\partial x}(k_{x}\frac{\partial T}{\partial x})+\frac{\partial }{\partial y}(k_{y}\frac{\partial T}{\partial y})+\frac{\partial }{\partial z}(k_{z}\frac{\partial T}{\partial z})+Q$$

In the above equation, ρ represents the density of the solid propellant; $C_{P}$ denotes the specific heat capacity; T and t represent temperature and time, respectively; $k_{i}$ signifies the thermal conductivity of the material in the three principal directions; and Q is the heat generated by the curing reaction per unit volume and time. The internal heat source Q is expressed as:

(2) $$Q=\rho H_{r}\frac{d\alpha }{dt}$$

where $H_{r}$ is the total heat released per unit mass during the curing reaction; dα/dt is the curing rate; and α represents the degree of cure. The actual charge filling process, including charging, pouring, and solidification of solid propellants, is performed in a constant-temperature environment; therefore, the first-class thermal boundary conditions can be applied to the outer surface of the housing. Boundary conditions for the first type of heat conduction:

(3) $$T=T_{a}$$

Under these natural convection conditions, $T_{a}$ represents the ambient environmental temperature, while T denotes the temperature at the object’s boundary, that is, the temperature specified at the boundary conditions.

### 2.3. Linear Thermoviscoelastic Constitutive Relations

To describe the viscoelastic behavior of the solid rocket motor, both the propellant and the insulation layer are treated as isotropic, linear viscoelastic materials. Under a three-dimensional stress state, the constitutive relationship is expressed using the following integral form [29]:

(4) $$\sigma_{ij}\left(t\right)=2\int_{-\infty }^{t}G\left(t-\tau \right)\frac{\partial \varepsilon_{ij}(\tau )}{\partial \tau }d\tau +\delta_{ij}\int_{-\infty }^{t}\lambda \left(t-\tau \right)\frac{\partial \varepsilon_{kk}(\tau )}{\partial \tau }d\tau$$

where t and τ denote time; σ and ε represent the stress tensor and strain tensor, respectively; G(t) is the shear relaxation modulus function; and λ(t) is the Lamé relaxation modulus function.

Because the grain undergoes significant temperature fluctuations during the curing and cooldown phases, the numerical model must account for the effects of thermal strain and the temperature sensitivity of the material’s mechanical properties. The resulting thermoviscoelastic integral constitutive [30] equation is as follows:

(5) $$\sigma_{ij}(t)=\;2\int_{0}^{t}G\left(\xi -\xi^{\prime }\right)\frac{de_{ij}(\tau )}{d\tau }d\tau +\delta_{ij}\int_{0}^{t}K\left(\xi -\xi^{\prime }\right)\frac{d}{d\tau }\left(\varepsilon_{kk}(\tau )-3\alpha \theta (\tau )\right)d\tau$$

In the above expression, ξ and $\xi^{\prime }$ are defined as the reduced equivalent times:

(6) $$\xi =\int_{0}^{l}\frac{d\tau }{\alpha_{r}(\tau )}\;\xi^{\prime }=\int_{0}^{\tau }\frac{d\tau^{\prime }}{\alpha_{T}(\tau^{\prime })}$$

The parameter $\alpha_{T}$ represents the temperature shift factor. Based on the time–temperature superposition principle for viscoelastic materials, this factor is characterized by the Williams-Landel-Ferry (WLF) equation [31]:

(7) $$\mathrm{lg}\alpha_{T}=-\frac{C_{1}(T-T_{r})}{C_{2}+(T-T_{r})}$$

### 2.4. Material Properties

The material parameters for the motor case, insulation layer, and solid propellant are summarized in Table 1. To accurately characterize the time-dependent mechanical response of the propellant, its elastic modulus is defined using a Pony series representation. Solid propellants are typical polymer composites exhibiting pronounced time- and temperature-dependent viscoelastic behavior. Consequently, they are modeled using a thermal viscoelastic constitutive model, with the temperature dependence of viscoelastic responses incorporated through the WLF time–temperature shift relationship. The propellant was regarded as a homogeneous and isotropic thermoviscoelastic material, and particle-scale heterogeneity, binder-particle debonding, microcrack initiation, and progressive damage evolution were not described.

### 2.5. Loading Conditions

To evaluate the structural integrity of the propellant grain under various operational environments, three primary loading scenarios were defined:

Case 1: Curing cooling, where the propellant grain was allowed to cool from the temperature of 58 °C to the temperature of 20 °C, thereby approximating the practical conditions of a solid rocket motor.

Case 2: Combined loading of curing-induced cooling and gravity.

Case 3: Rapid pressurization to an internal pressure of 8 MPa at a low ambient temperature of −45 °C.

### 2.6. Mesh Sensitivity Analysis

**Figure 2:**
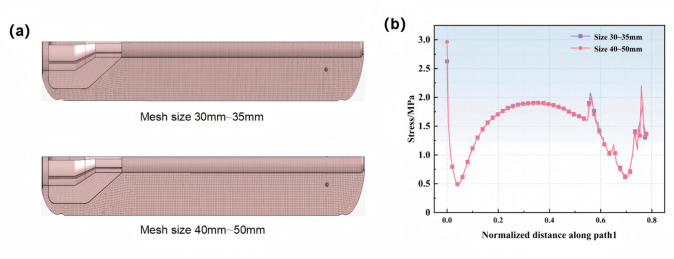
Verification of finite element mesh dependence size. (**a**) Solid propellant finite element models with different mesh size. (**b**) Stress variation curves under different mesh sizes.

To discuss the influence of mesh size on the analytical results, stress distributions along Feature Path 1 were analyzed using two different finite element mesh sizes, as shown in Figure 2a,b. The diagram illustrates the finite element models of solid propellant with varying mesh sizes, while the curves show stress variations under each condition. The nearly identical stress variation patterns across both mesh sizes demonstrate that a 40–50 mm mesh size meets the required computational accuracy standards. 

## 3. Results

### 3.1. Curing Cooldown Conditions

**Figure 3:**
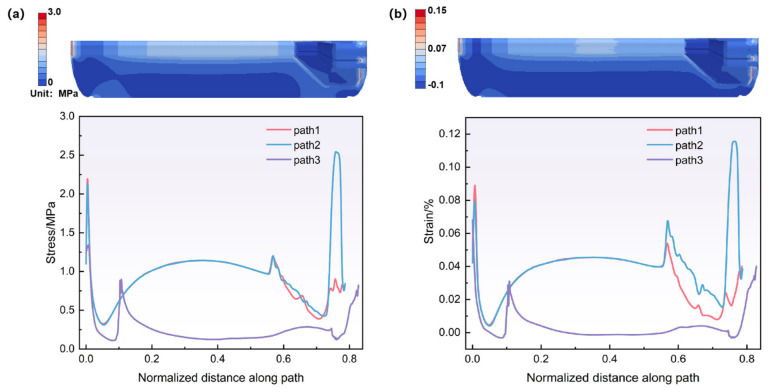
Response contours and characteristic-path curves of the defect-free propellant grain under the curing-induced cooling condition: (**a**) stress response contour and corresponding characteristic-path curves; and (**b**) strain response contour and corresponding characteristic-path curves.

Under the curing-cooling condition, the internal stress and strain responses of the propellant grain without voids showed pronounced dependence on the selected path and local geometric features. The stress evolution curves along Path 1 and Path 2 showed similar trends within the distance range of 0–0.6, both exhibiting an initial stress peak of approximately 2.0–2.2 MPa, followed by a rapid decrease to 0.3–0.4 MPa and a subsequent gradual increase to a plateau of about 1.1 Mpa, as illustrated in Figure 3a. This behavior reflects the initial stress concentration near the inner bore caused by thermal contraction during cooling; as the distance increases, the stress levels-off due to constraint release and structural transition. Near the normalized distance of 0.75, Path 2 exhibited a sharp stress increase with a peak greater than 2.5 MPa, exceeding the stress level of Path 1 at the corresponding position, suggesting that the wing-column end suffered a much stronger stress concentration than the wing-slot end during the cooling stage. In contrast, Path 3 exhibits a distinct stress distribution characterized by lower overall stress levels. A stress peak of 0.88 MPa occurs at the forward artificial debonding layer, while the stress remains consistently between 0.1 and 0.2 MPa in the middle segment. A slight stress recovery is observed at the slot-fin region, suggesting that the forward debonding area and the slot-fins are the most likely initiation sites for failure at the grain–insulation interface. As shown in Figure 3b, regarding strain distribution, Path 1 remains relatively stable, peaking at 0.09% near the front of the inner bore and maintaining a value of 0.04–0.05% through the middle segment, with a localized peak of 0.055% near the aft end. Path 2 shows more pronounced fluctuations, reaching a peak strain of 0.11% at the fin terminus. This suggests that the fin tip experiences a highly complex multiaxial stress state where viscoelastic relaxation is insufficient to offset the cumulative effects of structural mutation, marking it as a critical weak point in the structural integrity. Path 3 exhibits the lowest strain overall, as the flexible nature of the insulation layer effectively accommodates the thermal contraction of the grain, thereby mitigating strain at the interface. In summary, the high stress at the fin tips approaches the material’s elongation limit, identifying these areas and the forward debonding layers as primary regions of concern for engineering design.

**Figure 4:**
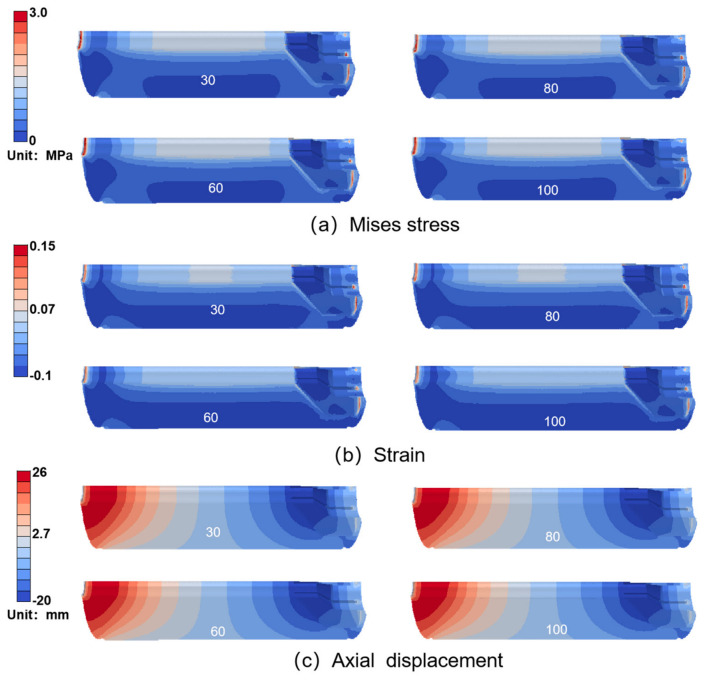
Structural response contours of propellant grains with different void defects: (**a**) stress distribution contour; (**b**) strain distribution contour; and (**c**) displacement distribution contour.

Figure 4 shows the structural response contours of propellant grains containing void defects of different sizes after curing-induced cooling. Figure 4a,b indicate that the grains exhibited almost identical mechanical response distributions under curing–cooling loading, with the maximum stress and strain occurring at the front end of the inner bore and the rear wing-slot region, respectively reaching 3.04 MPa and 0.148%. Figure 4c shows that larger displacements occurred at both ends of the grain compared with the remaining regions, because the front and rear artificial debonding layers made these end regions more susceptible to axial inward shrinkage. The maximum axial displacement occurred at the front end, with a value of approximately 26 mm.

**Figure 5:**
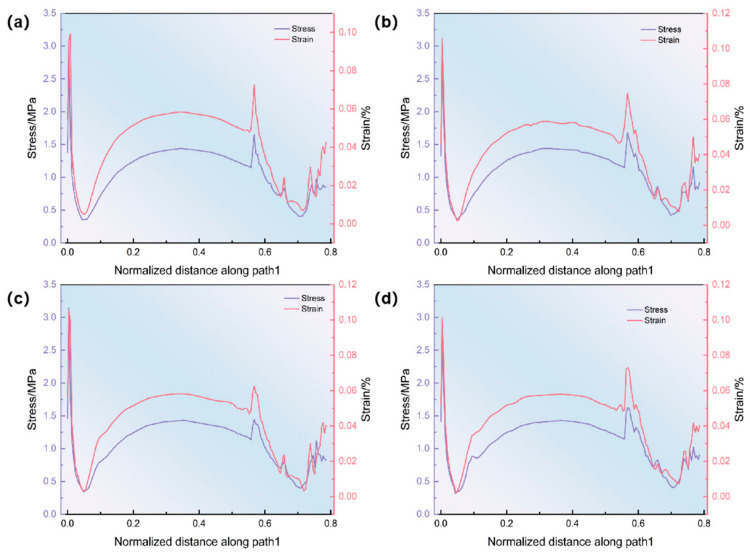
Stress and strain distributions along characteristic Path 1 for propellant grains with different void defects: (**a**) void radius of 30 mm; (**b**) void radius of 60 mm; (**c**) void radius of 80 mm; and (**d**) void radius of 100 mm.

Figure 5 shows the stress and strain profiles along characteristic Path 1 for the defect-free propellant grain and the grains containing void defects with different sizes. In general, the stress and strain variations along the characteristic path were broadly consistent for the grains containing voids with sizes ranging from 30 to 100 mm. In terms of magnitude, the grains with void defects showed higher overall stress and strain levels than the defect-free grain. The peak value is observed at the path’s starting point, resulting from the stress concentration effect at the pore edges. This initial peak stress tends to decrease as the pore size increases: for 30 mm and 60 mm pores, the peak stress is approximately 3.0 MPa; it decreases slightly at 80 mm and further drops to roughly 2.8 MPa for the 100 mm case. This phenomenon suggests that while small pores amplify stress concentration as localized defects, excessively large pores induce internal stress redistribution, which mitigates high-stress levels. At the path’s starting point, the strain across all pore sizes is approximately 0.1.

In the middle segment of the path, the stress and strain values were generally similar among the different void-size cases. This suggests that the void primarily affects a limited region near the defect tip, while the stress distribution in regions away from the defect is dominated by the global geometry of the grain and the applied loading condition. In the rear wing-slot region, the 80 mm void case showed the lowest response compared with the other void sizes, with a stress of 1.44 MPa and a strain of 0.062%. The wing slot is a geometric discontinuity, and when the void size is small, the void is relatively far from the wing slot; therefore, the superposition of void-induced stress concentration and geometric stress concentration at the wing slot leads to a significant increase in the stress peak in this region. When the void size increased to 80 mm, the spatial relationship between the void and the wing slot changed, and the interaction between their stress fields altered the stress transfer path, redistributing the stress originally concentrated at the wing slot to the material around the void, thereby effectively unloading the wing-slot region and significantly reducing the local stress. With a further increase in void size to 100 mm, the void extended beyond the effective interaction range of the stress fields, thereby offsetting the effect of stress redistribution and increasing the stress and strain responses. In practical engineering, a void radius of 80 mm is suggested as a reference control size, while the local load-bearing capacity around smaller voids should be enhanced to improve grain integrity through both void control and performance optimization.

**Figure 6:**
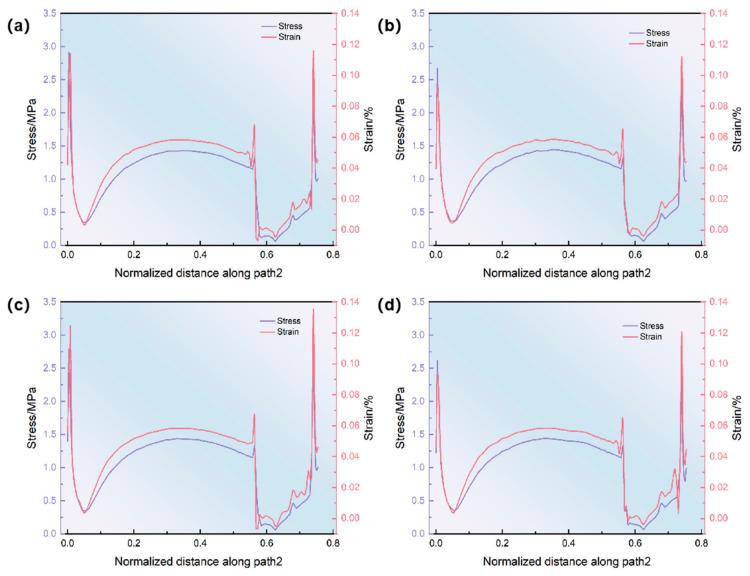
Stress and strain distributions along characteristic Path 2 for propellant grains with different void defects: (**a**) void radius of 30 mm; (**b**) void radius of 60 mm; (**c**) void radius of 80 mm; and (**d**) void radius of 100 mm.

Figure 6 illustrates the stress and strain distributions along characteristic Path 2 for drug columns with varying pore sizes. Along Path 2, the stress–strain distributions of samples with different pore sizes exhibit similar evolutionary patterns: a high local peak occurs at the path’s starting point, followed by rapid decline, then gradual recovery within the normalized distance range of 0.20–0.50, forming a relatively smooth distribution zone that indicates continuous stress transfer and stable deformation. When approaching the normalized distance range of 0.55–0.60, both stress and strain undergo significant abrupt changes, with strain peaks showing greater sensitivity, indicating substantial effects from pore defects and surrounding stress redistribution. As pore size increases from 30 mm to 100 mm, the overall stress profile in the central stable region does not follow a simple monotonic relationship; instead, stress and strain fluctuations intensify in the posterior path segment, particularly with pronounced localized strain concentration in larger pore models. This demonstrates that increased pore size expands the disturbance range of defects, compromises internal stress continuity within the drug column, and elevates risks of localized deformation concentration and structural integrity degradation.

#### 3.1.1. Interfacial Stress Response Between the Propellant Grain and Insulation Layer Under Curing-Induced Cooling

**Figure 7:**
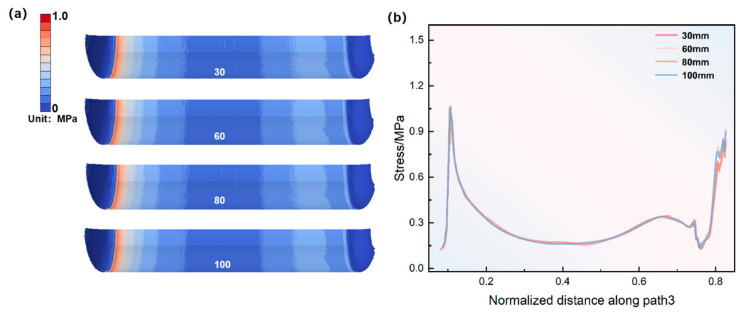
Stress response of the insulation layer and propellant/insulation interface under different void-size conditions: (**a**) stress response contours of the insulation layer; and (**b**) interfacial stress distribution along characteristic Path 3.

During motor operation, the propellant–insulation interface experiences significant mechanical loads, which pose a high risk of debonding. Because interface failure is primarily driven by stress, this section evaluates the interfacial stress response under curing cooldown conditions. Figure 7a illustrates the stress response contour plots for the insulation layer under these loads. The results demonstrate that the stress distribution within the insulation is nearly identical across models with different pore sizes. This indicates that the global stress profile of the insulation is largely independent of internal void dimensions, with the maximum stress consistently peaking at 0.81 MPa at the root of the forward artificial debonding layer.

Figure 7b depicts the distribution of interfacial equivalent stress along characteristic Path 3 during the curing cooldown phase. The stress magnitudes along this path remain numerically consistent across all pore sizes, further confirming the limited impact of internal defects on the overall interfacial stress distribution. The equivalent stress along Path 3 exhibits a fluctuating “increase–decrease–increase–decrease–increase” trend. This behavior is attributed to the combined effects of solidification shrinkage and external loading, resulting in two primary stress peaks: one at the root of the forward artificial debonding layer and another at the base of the slot-fins. Notably, the stress concentration is more severe at the forward artificial debonding layer, whereas the stress at the aft debonding layer root is relatively lower.

#### 3.1.2. Analysis of Propellant–Mandrel Contact Behavior During Curing-Induced Cooling

**Figure 8:**
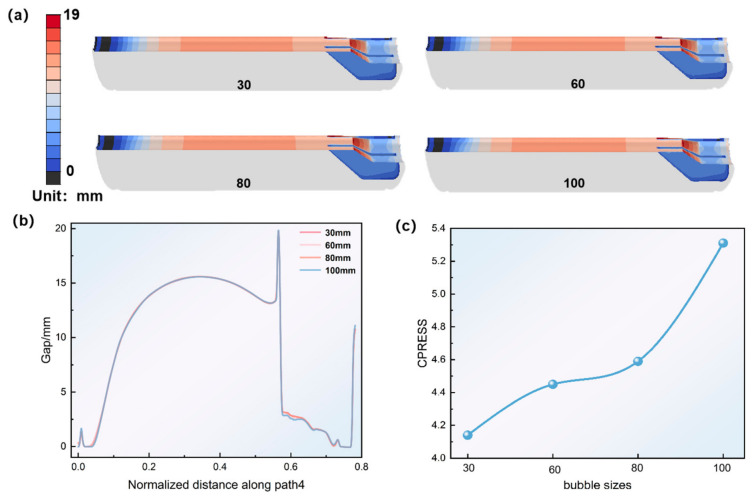
Numerical simulation results of propellant–mandrel contact behavior: (**a**) cloud map of the gap between the propellant and the core mold; (**b**) gap distribution along characteristic Path 4; and (**c**) variation of contact stress with void size.

To further investigate the evolution of residual stress in propellant grains containing void defects during curing-induced cooling, this section discusses the contact simulation between the propellant and the mandrel. As shown in Figure 8a, the contour responses are generally similar; relatively large distances between the propellant and the mandrel are observed at the variable cross-section of the wing-column region and in the middle section of the grain bore, indicating that a certain gap is maintained between the propellant and the mandrel. At the front end of the grain bore and the bottom of the wing slot, the gap between the propellant and the mandrel is nearly reduced to zero, and the black regions indicate that contact and compression have occurred.

The distribution curves along characteristic Path 4 illustrate the evolution of the contact behavior between the mandrel and propellant grains containing voids of different sizes during curing-induced cooling. As shown in Figure 8b, the gap variation trends of the grains with different void sizes are generally similar, with differences mainly occurring in the wing-slot region. At the front portion of Path 4, all curves show a transient and small gap, which then rapidly decreases to nearly zero. In the middle segment of Path 4, the gap increases rapidly, reaching its maximum at the junction between the grain bore and the wing slot, where the separation from the mandrel is the most pronounced during cooling shrinkage; the grain with a 100 mm void exhibits a slightly larger gap than those with 30–80 mm voids. The presence of pores significantly reduces the structural stiffness of the grain, making its separation from the mandrel more pronounced during cooldown. Conversely, regions near smaller pores experience stronger constraints, which restricts the degrees of freedom for thermal contraction.

When the void size is 30 mm, the contact pressure is approximately 4.14 MPa. As the pore size increases to 60 mm, 80 mm, and 100 mm, the contact pressure rises to 4.45 MPa, 4.659 MPa, and 5.31 MPa, respectively. In all instances, the peak contact pressure is situated at the base of the slot-fins. These findings indicate that increasing pore size alters the structural stiffness and thermal contraction of the grain, thereby influencing the contact state with the mandrel. During the curing cooldown process, the mandrel constrains grain contraction. Larger pores intensify local stress concentrations, leading to higher interfacial contact pressure, which ultimately increases the difficulty of successfully demolding the grain from the mandrel.

### 3.2. Combined Curing Cooldown and Gravitational Loads

#### 3.2.1. Influence of Coupled Curing Cooling and Gravitational Loading on the Stress–Strain Response of the Propellant Grain

Gravity, as a continuous load, cannot be neglected during the incompletely cured stage of the propellant or during storage and transportation after curing. During the casting–curing process, the uncured or partially cured slurry may experience microscale settlement under gravity, which further enhances the heterogeneity of the internal stress field. This gravitational effect superimposes with the thermal stresses generated during cooldown, resulting in complex stress fields within the grain—particularly in larger configurations or those with intricate geometries.

**Figure 9:**
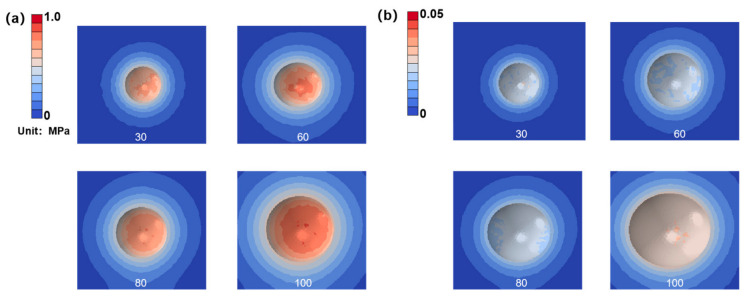
Stress–strain distributions around void defects under coupled curing cooling and gravitational loading: (**a**) stress distribution contour; and (**b**) strain distribution contour.

The stress and strain contours for propellant grains containing four distinct pore radii under combined curing cooldown and gravity loads are illustrated in Figure 9. As shown in the stress contours (Figure 9a), stress levels in the vicinity of the pores are significantly higher than in the surrounding matrix. Furthermore, both the intensity and extent of these high-stress regions expand as the pore size increases, with the maximum stress reaching 0.838 MPa at the 100 mm pore. For larger void radii, such as 80 mm and 100 mm, the external loading induces a higher pressure on the inner wall of the void. As internal geometric discontinuities, pores provide no structural support during thermal contraction, leading to severe localized stress concentrations. The introduction of gravitational loads further exacerbates this non-uniformity, making the stress at the pore boundaries even more pronounced. In the strain contours (Figure 9b), strain is primarily concentrated at the pore margins, with the distribution range and magnitude increasing proportionally with defect size. Because localized stress concentration directly induces higher strains, larger pores cause more substantial disruptions to structural continuity. Under identical thermal and gravitational loads, larger pores necessitate greater localized deformation to accommodate the stress field, resulting in a maximum strain of 0.025% at the pore. Under these combined conditions, the peak stress and strain values within the entire grain assembly are confined exclusively to the pore regions. The presence of these internal defects compromises material homogeneity, preventing uniform stress transmission and causing load accumulation at the void boundaries. Gravity imposes additional compressive deformation on the pore walls, rendering these voids the most critical structural vulnerabilities. In contrast, the continuous segments of the grain exhibit relatively uniform stress distributions with significantly lower numerical values. In subsequent propellant design, the influence of void size on structural integrity should be carefully balanced to ensure material reliability in practical applications.

#### 3.2.2. Analysis of Propellant–Mandrel Contact Behavior Under Coupled Curing Cooling and Gravitational Loading

**Figure 10:**
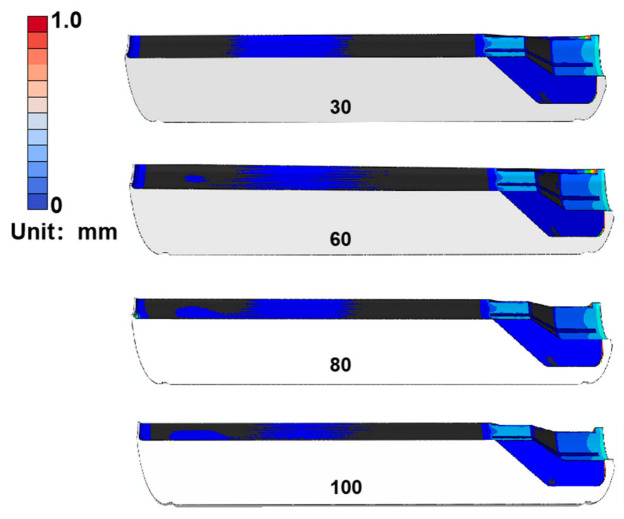
Contour plot of propellant–mandrel contact behavior.

As illustrated in Figure 10, the application of gravitational loads shifts the location of the maximum interfacial gap between the propellant and the mandrel. While the structural responses across all simulated cases remain fundamentally consistent, the most significant separation occurs at the base of the slot-fins, where the gap measures approximately 0.93 mm. Furthermore, due to the effect of gravity, the gaps at the forward and aft ends of the propellant bore, as well as along the middle section of the grain fins, are reduced to nearly zero.

### 3.3. Internal Pressure Loading

**Figure 11:**
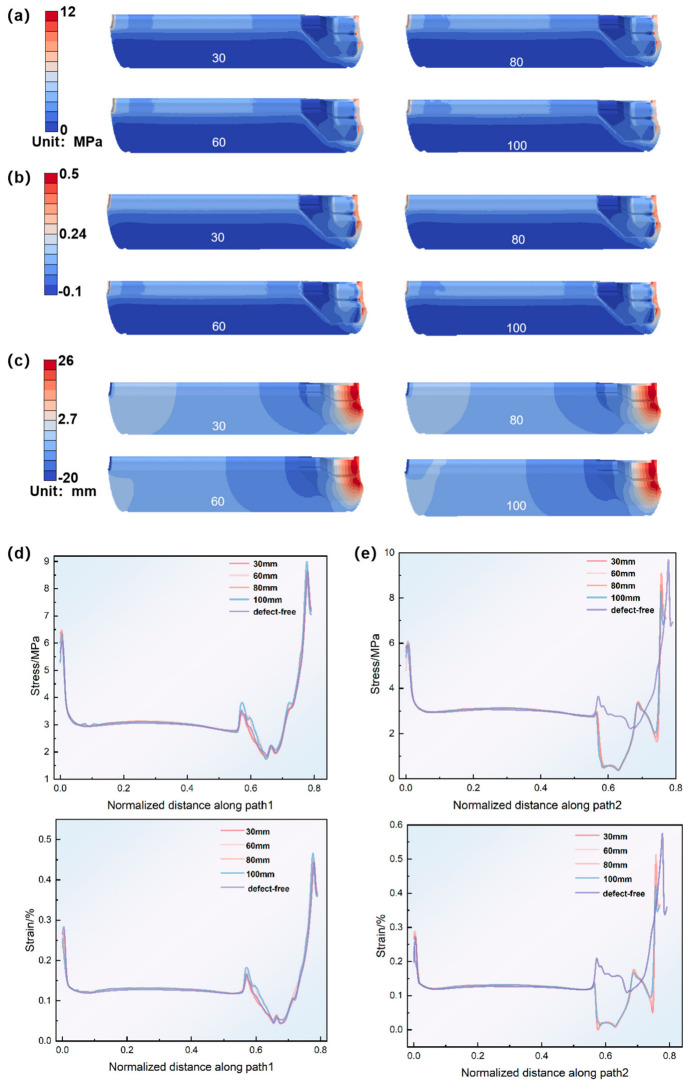
Structural response contours and characteristic-path distributions of the propellant grain under internal pressure loading. (**a**) stress distribution contour; (**b**) strain distribution contour; (**c**) displacement distribution contour; (**d**) stress and strain profiles along characteristic Path 1; and (**e**) stress and strain profiles along characteristic Path 2.

Figure 11a–c shows the structural response contours of propellant grains with different void defects and without defects under internal pressure loading. As shown in Figure 11a,b, the stress and strain values of the propellant grains with void sizes of 30–100 mm are generally similar. As observed in Figure 11c, under internal pressure loading, the grain shows an overall downward displacement compared with the curing-induced cooling condition, characterized by increased displacement at the rear end and decreased displacement at the front end.

Under internal pressure loading, porous and defect-free grains exhibit similar global behavioral patterns, though significant localized discrepancies are observed. The stress and strain distributions along the two characteristic paths follow a consistent profile: an initial peak at the starting segment, a stable plateau in the middle, localized fluctuations at transition zones, and a sharp escalation at the terminus. Along both Path 1 and Path 2, stress and strain peak at the initiation point before stabilizing into a steady-state region. In Path 1, stress levels in the central bore segment are approximately 3 MPa with a stable strain of 0.12% across all models, indicating that internal pores have a negligible influence on this region. However, pronounced fluctuations emerge at the transition between the bore and the slot-fins, as well as at the base of the slot-fins. For the grain containing a 100 mm pore, both stress and strain reach their maxima: peak stresses are recorded at 3.83 MPa and 8.97 MPa, while strain peaks reach 0.18% and 0.464%. This suggests that large-scale pores significantly compromise the material’s resistance to deformation, causing loads to accumulate at geometric discontinuities and exacerbating localized structural damage. At the transition zone of Path 2, the defect-free grain acts as a continuous structure where internal pressure is transmitted directly, resulting in high stress and significant deformation. Conversely, porous grains exhibit a sharp decline in stress—dropping to a minimum of approximately 0.568 MPa—with a concurrent reduction in strain. This “unloading effect” occurs because the pore redirects the stress transmission path, forcing the load to bypass the defect and effectively shielding the adjacent material from the primary load. In the defect-free case, the strain response is more complex, with peaks of approximately 0.22% at the transition zone and 0.582% at the slot-fin base.

The stress and strain reach their peak values at the terminal segment of the path. The end of the wing slot may represent a critical failure-prone region under the internal pressure condition. The void defects exert limited influence on the middle portions of the characteristic paths, whereas they markedly affect the transition region and the wing-slot end, indicating a response feature of global similarity and local amplification.

## 4. Discussion

This study analyzed the structural response of propellant grains containing voids of different sizes under three loading conditions: curing-induced cooling, coupled curing-induced cooling and gravity, and low-temperature internal pressure. The results show that the void acts as an internal geometric discontinuity in the propellant grain. This discontinuity disrupts structural continuity and changes the local stress-transfer path. As a result, the void induces stress redistribution and local response amplification. As the void size increases, its influence gradually extends to the wing slot, the bore-to-wing-slot transition region, and the contact region between the propellant and the mandrel. This indicates that void size is a key parameter affecting the structural integrity of the propellant grain.

Under curing-induced cooling, the structural response of the grain is mainly governed by thermal shrinkage, geometric constraints, and local structural discontinuities. The stress and strain distributions of the defect-free grain show clear path dependence and geometric sensitivity. The wing-column end, the front artificial debonding region, and the bore-to-wing-slot transition region are the main weak regions. These regions are prone to stress concentration. After the void is introduced, the mechanical response of the grain changes significantly. This change shows a clear size-dependent effect. When the void size is 30 mm or 60 mm, the void mainly acts as a local stress concentration source. When the void size increases to 80 mm, the void-induced stress field interacts with the stress field near the weak structural region. This stress-field interaction causes part of the stress to be redistributed. Therefore, the stress level in the wing-slot region is reduced to some extent. This stress-field interaction causes part of the stress to be redistributed. When the void size further increases to 100 mm, the larger cavity weakens the local load-bearing capacity of the grain. In this case, the large void offsets the previous stress-relief effect and increases the local stress level. In addition, increasing void size raises the contact pressure between the propellant grain and the mandrel. This increases the difficulty of demolding after curing shrinkage. By contrast, the void size has a limited influence on the overall stress distribution at the propellant/insulation interface. The interface stress is mainly concentrated at the root of the front artificial debonding layer and at the bottom of the wing slot. This indicates that the critical interface locations are mainly controlled by the overall structural constraints and the configuration of the artificial debonding layer.

Under the coupled effect of curing-induced cooling and gravity, stress and strain concentration near the void is further intensified. The asymmetric deformation caused by gravity is superimposed on the thermal shrinkage stress. This coupled effect changes the continuity of stress transfer inside the grain. Therefore, the defect effect of large voids becomes more pronounced. The void boundary becomes the main structural weak region under this loading condition and affects the structural continuity and integrity of the propellant grain. Under low-temperature internal pressure loading, the stress and strain in the middle section of the bore remain relatively stable. The stress in this region is approximately 3 MPa, and the strain is approximately 0.12%. This indicates that the void has a limited influence on regions far from the defect and geometric discontinuities. However, obvious fluctuations and local peaks in stress and strain occur at the bore-to-wing-slot transition region and at the bottom of the wing slot. This phenomenon is more pronounced in the model with a 100 mm void. These results suggest that the critical regions under internal pressure loading are mainly governed by geometric discontinuity, void-induced load bypassing, and local stiffness degradation. This finding is consistent with previous studies on the structural integrity of propellant grains under low-temperature ignition and internal pressure loading, in which transition sections, wing-slot regions, and geometric discontinuities were identified as stress concentration regions.

The applicability of the finite element model was further evaluated from the perspective of the modelling framework and comparison with published studies. The adopted thermoviscoelastic finite element framework adopted in this study is consistent with the pronounced time- and temperature-dependent viscoelastic behavior of solid propellants [34]. The temperature dependence of the viscoelastic response was described using the WLF time–temperature shift relation [7]. This method has been widely used in previous structural integrity analyses of solid propellant grains.

The present results were then compared with published studies on the structural integrity of solid propellant grains under low-temperature ignition and internal pressure loading. Wang Jiaqi et al. [35,36] investigated the structural response of a propellant grain under the combined loading of −40 °C and 10.4 MPa ignition pressure. Their results showed that the maximum Von Mises strain occurred at the junction between the bottom transition section and the cylindrical section of the grain. They also reported a safety factor of 2.46, indicating that the structural integrity requirement was satisfied. In the present study, the stress and strain in the middle section of the bore remain stable under internal pressure loading, whereas clear stress and strain concentrations appear at the bore-to-wing-slot transition region and at the bottom of the wing slot. This is consistent with the conclusion that geometric transition regions are prone to stress concentration under low-temperature internal pressure loading. In addition, previous studies have shown that local compression between the propellant and the mandrel can increase the Von Mises stress, contact stress, and contact area of the propellant. The present results show a similar tendency. During curing-induced cooling, the presence of void defects changes the local stiffness and stress-transfer path of the grain. As the void size increases, the contact pressure between the propellant grain and the mandrel increases, indicating that internal defects can aggravate local compression and affect the demolding process. Zhang Yuan et al. [37] investigated the structural integrity of propellant grains with different scaling ratios. The present results are consistent with their conclusions. Under curing-induced cooling, the void mainly causes local stress concentration and has a limited influence on the overall stress distribution at the propellant/insulation interface. Under coupled curing-induced cooling and gravity, the void boundary becomes a structurally weak region. Under internal pressure loading, the response in the middle section of the bore remains stable, while stress concentration occurs at the bore-to-wing-slot transition region and at the bottom of the wing slot.

The high-stress regions of the model align with the typical critical areas identified in experimental numerical studies of solid propellant charges under thermal and pressure loads [38,39]. Published X-ray and CT studies have also confirmed that pores and voids are typical internal defects in solid propellants. These defects may act as geometric discontinuities and local stress concentration sources [4]. Remakanthan et al. [40] reported that X-ray radiography is an effective non-destructive testing method for identifying internal defects in solid rocket motor grains. This provides a physical basis for treating the void as an internal geometric discontinuity and a potential stress concentration source in the present model. In addition, in situ X-ray tomography studies of solid propellants have shown that the matrix, particles, and internal defects can be clearly identified during loading. Tang et al. [41] reviewed recent advances in the structural reliability analysis of solid propellant grains and pointed out that curing shrinkage, internal pressure, deformation mismatch, and complex grain geometry are key factors affecting grain deformation and structural integrity. Therefore, the stress distribution trends and critical regions predicted in this study are consistent with available experimental observations, X-ray tomography measurements, cold-pressurization experimental–numerical comparisons, and structural reliability studies [37]. These comparisons indicate that the present numerical model can reasonably capture the stress redistribution induced by void defects in solid propellant grains, including void-induced discontinuity, local stress concentration, and response amplification near geometric transition regions.

Overall, void defects are important factors affecting the structural integrity of propellant grains. The wing-column end, the bottom of the wing slot, the bore-to-wing-slot transition region, and the root of the artificial debonding layer should be regarded as weak regions. In engineering applications, reinforcement design should be considered for these weak geometric transition regions to improve the safety margin of the motor during storage and operation.

## 5. Conclusions

This study establishes a three-dimensional finite element model of the propellant–insulation layer–mandrel to investigate the structural response of propellant grains containing void defects of different sizes. Numerical simulations were carried out for the defect-free grain and grains containing void defects ranging from 30 mm to 100 mm under three representative service conditions, namely Curing Cooldown Conditions, coupled curing-induced cooling and gravity, and internal pressure loading. The stress–strain distribution within the propellant grain, the mechanical response at the propellant/insulation interface, and the contact behavior between the propellant and the mandrel were systematically analyzed. The main conclusions are summarized as follows:

Under Curing Cooldown Conditions, void defects change the local stress-transfer path of the propellant grain and induce stress concentration. The 30 mm and 60 mm voids mainly lead to local stress amplification. For the 80 mm void, the interaction between the void-induced stress field and the stress field near the wing-slot region partially redistributes the local stress, thereby reducing the stress level in the wing-slot region to some extent. In contrast, the 100 mm void significantly weakens the local load-bearing capacity of the grain and increases the risk to structural integrity. As the void size increases, the contact pressure between the propellant grain and the mandrel also increases, indicating greater difficulty during demolding. By comparison, the void size has only a limited influence on the overall stress distribution at the propellant/insulation interface, where the critical regions are mainly located at the root of the front artificial debonding layer and the bottom of the wing slot.

The combined curing cooldown and gravitational loads further amplifies the defect-induced mechanical response near the void. The asymmetric deformation caused by gravity, together with the residual stress generated during curing-induced cooling, makes it difficult for the load to be transferred continuously and uniformly within the structure of a solid propellant grain. As a result, stress and strain are mainly concentrated around the void boundary. With increasing void size, both the magnitude and the spatial extent of the high-stress and high-strain regions around the void increase. For the 100 mm void, the maximum stress and strain at the void boundary reach 0.838 MPa and 0.025%, respectively, indicating that large void defects become the most critical weak regions under this loading condition.

Under internal pressure loading, the void defects have a limited influence on the stress and strain in the middle section of the bore. The stress in this region remains approximately 3 MPa, and the strain remains approximately 0.12%, indicating a relatively stable global response. However, the presence of void defects intensifies local stress concentration at the bore-to-wing-slot transition region and at the bottom of the wing slot. This exhibits distinct characteristics of “overall similarity with localized enlargement”.

Comparisons with published studies on low-temperature ignition, non-destructive testing, and X-ray tomography indicate that the critical regions predicted in this study are consistent with the geometric transition regions and internal defect-sensitive zones reported in the literature. This agreement suggests that the proposed finite element model can reasonably capture the relative influence of void size on the structural integrity of solid propellant grains. Nevertheless, further experimental validation using grains with controlled void defects and CT-reconstructed models is still required to improve the quantitative prediction of failure load and service life.

## Figures and Tables

**Table 1:** Material parameters of the motor components [32,33].

Parameter	Propellant	Insulation	Case
$\mathrm{Density}/(\mathrm{kg}\cdot m^{-3})$	1800	1720	8000
Poisson ratio	0.495	0.49	—
$\mathrm{Specific}\;\mathrm{heat}/(J\cdot {\left(\mathrm{kg}\cdot K\right)}^{-1})$	1860	1820	500
$\mathrm{Thermal}\;\mathrm{conductivity}/(W\cdot {\left(m\cdot K\right)}^{-1})$	0.32	0.162	42
${\mathrm{Expansion}\;\mathrm{coefficient}/K}^{-1}$	0.00015	1.73 × 10^−5^	1.73 × 10^−5^
Modulus/MPa	0.7	1.20	—

## Data Availability

The original contributions presented in this study are included in the article. Further inquiries can be directed to the corresponding author.
